# Symmetry of the left and right tibial plafond; a comparison of 75 distal tibia pairs

**DOI:** 10.1007/s00068-024-02568-x

**Published:** 2024-06-14

**Authors:** Joy Verbakel, Miriam R. Boot, Nynke van der Gaast, Hans Dunning, Max Bakker, Ruurd L. Jaarsma, Job N. Doornberg, Michael J. R. Edwards, Sebastiaan A. W. van de Groes, Erik Hermans

**Affiliations:** 1 Department of Trauma Surgery, Radboud University Medical Center, Geert Grooteplein Zuid, 6525 GA Nijmegen, The Netherlands; 2 Orthopaedic Research Laboratory, Radboud University Medical Center, Nijmegen, The Netherlands; 3 Department of Orthopaedic Surgery, Radboud University Medical Center, Nijmegen, The Netherlands; 4 Department of Orthopaedic & Trauma Surgery, Flinders University and Flinders Medical Centre, Adelaide, Australia; 5 Department of Orthopaedic Surgery, University Medical Center Groningen, Groningen, The Netherlands

**Keywords:** Tibial plafond, Tibial plafond fractures, Pilon fractures, Symmetry, 3D virtual planning, Surgical planning

## Abstract

**Purpose:**

Tibia plafond or pilon fractures present a high level of complexity, making their surgical management challenging. Three-Dimensional Virtual Planning (3DVP) can assist in preoperative planning to achieve optimal fracture reduction. This study aimed to assess the symmetry of the left and right tibial plafond and whether left–right mirroring can reliably be used.

**Methods:**

Bilateral CT scans of the lower limbs of 75 patients without ankle problems or prior fractures of the lower limb were included. The CT images were segmented to create 3D surface models of the tibia. Subsequently, the left tibial models were mirrored and superimposed onto the right tibia models using a Coherent Point Drift surface matching algorithm. The tibias were then cut to create bone models of the distal tibia with a height of 30 mm, and correspondence points were established. The Euclidean distance was calculated between correspondence points and visualized in a boxplot and heatmaps. The articulating surface was selected as a region of interest.

**Results:**

The median left–right difference was 0.57 mm (IQR, 0.38 – 0.85 mm) of the entire tibial plafond and 0.53 mm (IQR, 0.37 – 0.76 mm) of the articulating surface. The area with the greatest left–right differences were the medial malleoli and the anterior tubercle of the tibial plafond.

**Conclusion:**

The tibial plafond exhibits a high degree of bilateral symmetry. Therefore, the mirrored unfractured tibial plafond may be used as a template to optimize preoperative surgical reduction using 3DVP techniques in patients with pilon fractures.

## Introduction

Fractures of the tibial plafond, or pilon fractures, are relatively rare and account for < 1% of all lower extremity fractures [1, 2]. Most fractures result from high-energy trauma (HET) mechanisms, such as skiing- and motorcycle injuries and falls from height [3, 4]. Additionally, low-energy trauma mechanisms are associated with osteoporosis in the elderly [2]. Fractures of the tibial plafond do occur in patterns [5], but are complex intra-articular fractures which can permanently affect the ankle joint, therefore, these fractures nearly always require surgical fixation, or even direct arthrodesis in cases beyond repair [6]. Due to the complex configuration of these fractures, surgical treatment can be challenging. Patients after a HET commonly suffer from severe soft tissue damage, making patients highly susceptible to complications such as infections, posttraumatic osteoarthritis, non- or mal-union and their long-term functional outcomes show poor results, both physical and psychological [4, 7, 8].

Recognition and understanding of the fracture and its fracture lines are crucial for determining the optimal surgical approach for fracture reduction, in order to regain anatomical restoration and stability of the joint [5, 9]. It has been recognized that optimal anatomic reconstruction of the ankle is key to prevent these complications and improve patient outcomes [9, 10]. 2DCT and 3D CT scans are the cornerstone of pre-operative planning, and related to clinical preoperative radiographs to visually identify the fragments in need of reduction or manipulation using Image Intensifier (II) during the procedure [11, 12]. Recognition of fracture pattern is important for the patients’ prognosis, for surgeons could gain a more thorough understanding of the fragments and therefore create a suitable preoperational plan [13–15].

Three-Dimensional Virtual Planning (3DVP) is an emerging technology in orthopaedic trauma [16–18]. Various studies evaluating 3D virtual software and 3D printed models in preoperative planning of complex fractures show a reduction in operation time, blood loss and number of fluoroscopies [19–25]. Using 3DVP, the contralateral, non-fractured, tibial plafond could be used as a template for optimal reduction of the fracture. However, there is a lack of evidence that the differences between both tibial plafonds are sufficiently small to use the contralateral tibial plafond as a template for 3DVP. Several studies have been performed on assessing limb symmetry using different methods [26–30]. For example, Tümer et al. [28], used the whole tibia to compare left with right within the ankle joint. However, they did not specifically focus on the tibial plafond, but mostly on the inter-subject fibula and tibia diameter which can influence the difference in symmetry. A study of Gabrielli et al. [29] found that there are no differences for tibial plafonds within a group of twenty healthy individuals using statistical shape modelling. Even though they created a good comparison between the left and right ankle for those twenty patients, their work however is less indicative for the left–right symmetry within one patient.

Therefore, this study aimed to investigate the similarity of the left and right tibial plafond and to assess whether the contralateral side of the tibial plafond can be safely used to optimize the preoperative surgical reduction using 3DVP. We hypothesized that there are no significant differences between the left and right tibial plafond.

## Methods

Data for this study was collected by a previous imaging study in which 109 participants underwent a CT angiography (CTa) scan of their left and right tibia with a slice thickness of 1 mm and average voxel size of 0.791 × 0.791 × 0.768 mm (Canon Aquilion One). The study was approved by the local Ethics committee and all subjects provided consent for secondary use of the data. The data of patients with two comparable ankle joints and available CT images of the whole tibia with a slice thickness of 1 mm or less were included in the analysis. After excluding patients with previous ankle surgery, severe ankle trauma, ankles with visible signs of osteoarthritis, CT scans with incomplete visibility of either of the tibias, a voxel size of > 1 mm, and excessive bone density differences between the left and right tibia, impeding automatic segmentation. 75 CT images were included.

The left and right tibia of all 75 CT images were segmented using a convolutional neural network (CNN) [31] and afterwards manually adjusted in 3D slicer (5.0.3, Brigham and Women’s Hospital, Boston, Massachuettes, United States). The segmentations were converted to 3D bone models after which the mesh quality was improved automatically using MATLAB (The MathWorks Inc, Natick, Massachusetts, United States).

**Fig. 1:**
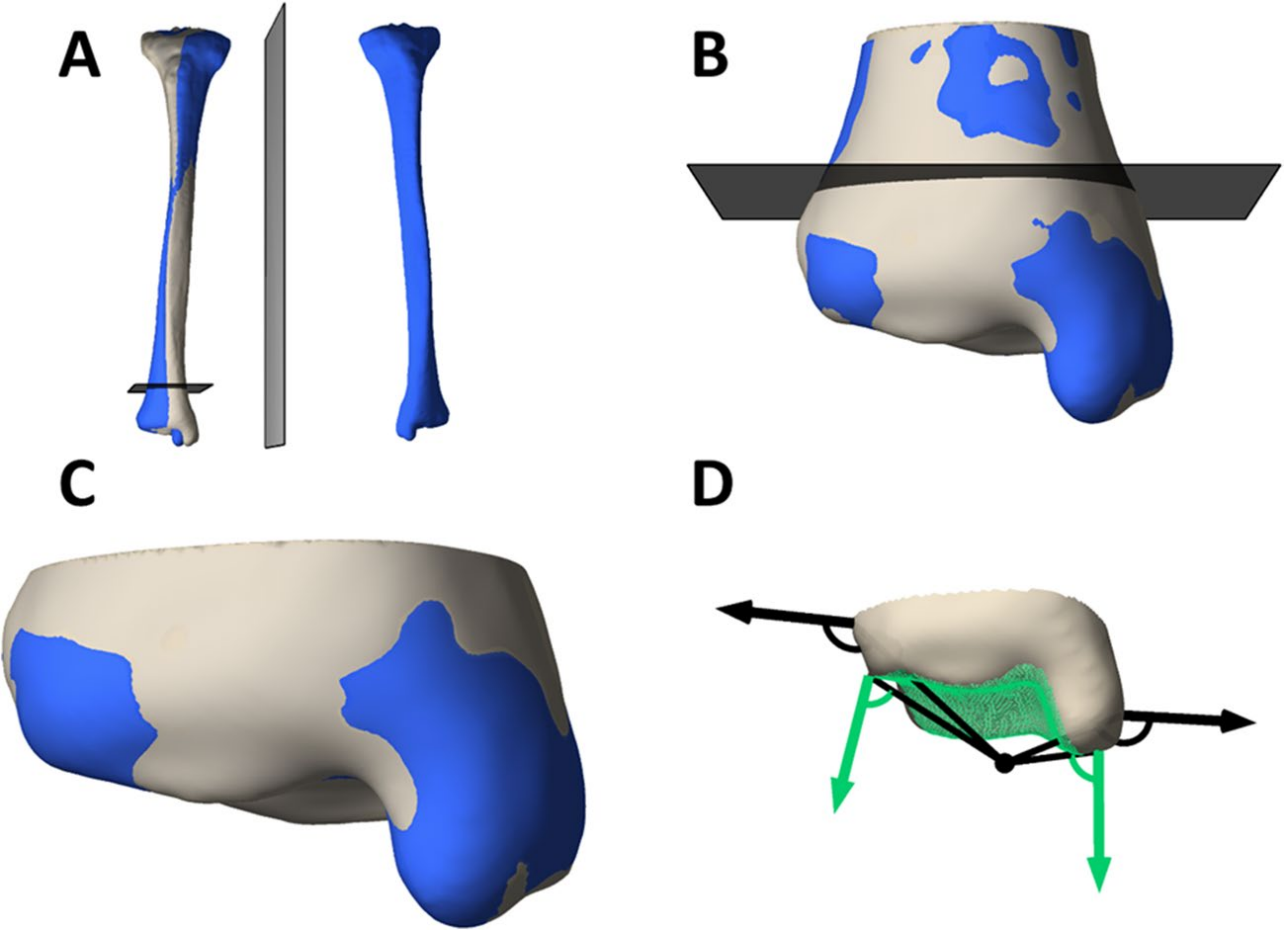
Extraction of correspondence points to compare the symmetry of the left and right tibial plafond in one participant. **A** Bone models were constructed of the right and left tibia, after which the left tibia (blue) was mirrored along the sagittal plane and superimposed on the right tibia (bony color). **B** Both tibias were cut 50 mm above the most distal point of the medial malleolus and superimposed again. **C** A second cut was made followed by a final superposition step to obtain tibial plafonds with a height of 30 mm. **D** The articulating surface was selected as a specific region of interest by comparing the angle between each vertex normal and the line connecting each vertex and the reference point. Angles less than 90° were considered as articulating surface (green arrows)

To assess the left–right symmetry of the tibia plafond, the left tibias were mirrored in the sagittal plane. The mirrored left tibias were superimposed on the contralateral right tibias using a computer-based rigid Coherent Point Drift (CPD) surface matching algorithm [32]. Subsequently, the right and superimposed mirrored left tibias were cut at 50 mm and at 30 mm above the most distal point of the medial malleolus, and were superimposed each time to derive two equally sized tibial plafonds with a height of 30 mm. These incremental cutting and superposition steps ensured optimal registration of the tibial plafond (Fig. 1). Correspondence points were established at the most distal 25 mm of the tibial plafond, using nonrigid point set registration with the CPD algorithm of the right bone models onto the contralateral superimposed mirrored left tibias. This way, the distance between the corresponding points measured the distance between the surfaces. The articulating surfaces were selected as a specific region of interest. To do so, the right tibias were rotated into the anatomical reference directions to calculate a reference point at the center of the distal tibia, at the height of the most distal point on the medial malleolus [33]. The center of the distal tibia was established as the mean anteroposterior and mediolateral coordinate of the vertices describing the distal tibia. Subsequently, all vertices facing the reference point were determined as the articulating surface, by comparing the angles between the vertex normal with the line connecting each vertex and the reference point. Angles smaller than 90° were considered as articulating surface. The Euclidean distance was calculated between the correspondence points of the entire tibial plafond and the correspondence points of the articulating surfaces only. The normality of the data was visually checked with a histogram. As the data did not meet the assumption of normality, the distance of the correspondence points was summarized in boxplots and visualized with heatmaps using MATLAB.

## Results

**Fig. 2:**
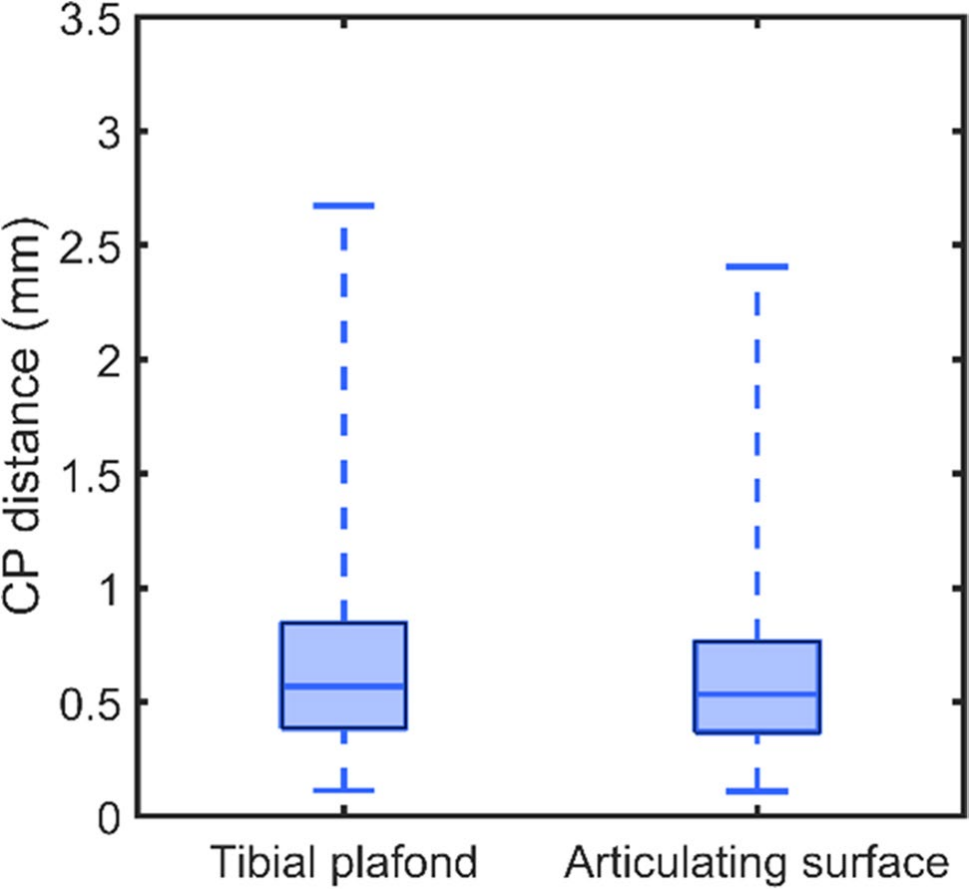
Boxplot showing the Euclidean distance between the left and right correspondence points (CP) of the 25 mm tibial plafond regions and articulating surfaces. The whiskers extend from the 1st to the 99th percentile

**Fig. 3:**
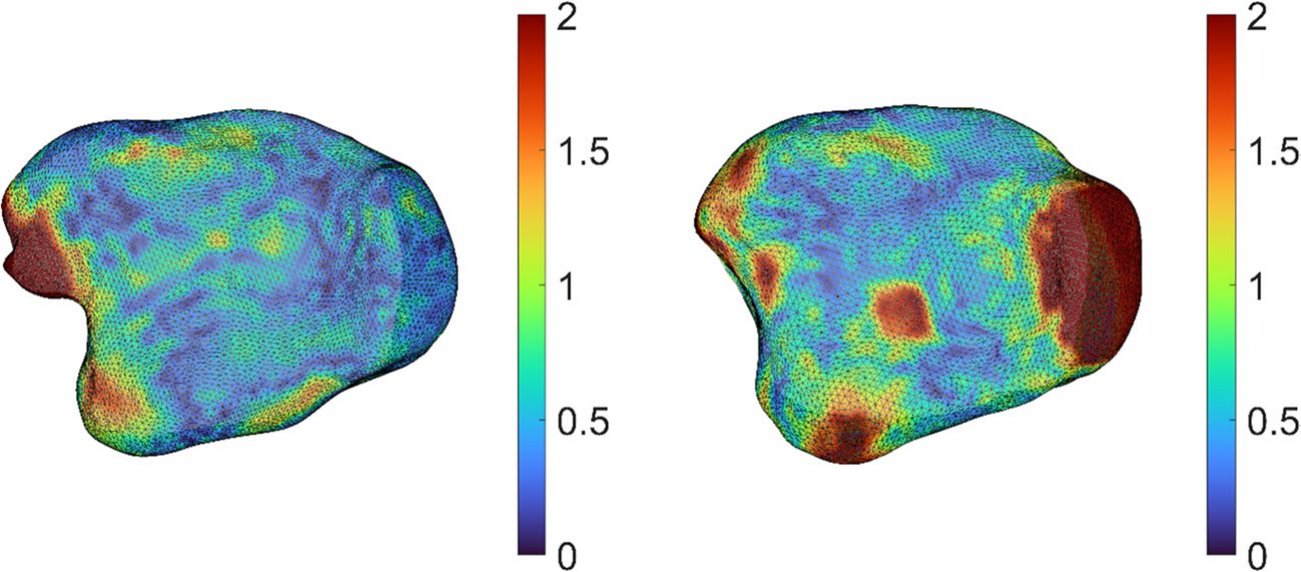
Heatmap showing the axial view of the left–right symmetry of the tibial plafond for the two participants with the largest maximal distance of the correspondence points. The heatmaps were based on the Euclidean distance between the left and right correspondence points and displayed on the right tibial plafond. The color bar displays the distance in mm. Distances > 2 mm are displayed in dark red

The median Euclidean distance of the correspondence points of the entire tibial plafond was 0.57 mm (IQR, 0.38 – 0.85) and the articulating surface was 0.53 mm (IQR, 0.37 – 0.76; Fig. 2). The largest differences between the correspondence points were typically seen at the medial malleolus and the anterior tubercle of the tibial plafond (Fig. 3). The overall maximal distance between the correspondence points was 8.9 mm.

## Discussion

The aim of this study was to evaluate the left and right differences of the tibial plafond using CT scans of ankles without a previous injury, to determine whether left–right mirroring can be safely used to optimize preoperative 3D virtual planning for patients with pilon fractures.

This study shows that the tibial plafond is highly symmetrical as the median distance of the correspondence points was 0.57 mm (IQR, 0.38 – 0.85) for the entire tibial plafond and 0.53 mm (IQR, 0.37 – 0.76) for the articulating surface. Given these small distances, this study confirms that the contralateral tibial plafond can reliably be used as a template when treating pilon fractures. The greatest distances between corresponding points were primarily found in the medial malleolus and the anterior tubercle of the tibial plafond, with a maximum of 8.9 mm (Fig. 3). This participant did not suffer previous trauma to the tibial plafond. It is possible that the tibial plafonds portray the most significant asymmetry at these points, although this asymmetry might stem from inaccuracies in the segmentation process. The limited contrast of the medial malleolus and the anterior tubercle of the tibial plafond made it difficult to accurately differentiate between soft tissue and bone. As a result, a region could be marked as soft tissue in one tibial plafond and bone in the contralateral side. For example, the prominent part of the anterior tubercle of the tibial plafond visible in Fig. 3 may partly be the result of segmentation errors.

In accordance with the present results, previous studies have mostly demonstrated that healthy human limbs exhibit bilateral symmetry, observing left–right differences in the same order of magnitude. Van der Gaast et al. [34] showed that the tibial plateau is highly symmetric with an average left–right difference in correspondence points of 0.6 mm and concluded that the contralateral tibial plateau can safely serve as a template in 3D virtual preoperative planning. Likewise, a study of Letta et al. [35] shows high correlation between the left and right scaphoid in three dimensions with respect to volume, surface area and length. The average discrepancy was 0.26 mm, with the largest difference being 1.9 mm. Moreover, Islam et al. [36] demonstrated that the left and right talus bones have a strong degree of symmetry with a mean left–right deviation of -0.74 to 0.62 mm.

In addition to these findings, previous research on the distal tibia also supports our findings of bilateral symmetry. Kellam et al. [37] examined the plafond radius of curvature in 215 radiographs and found no significant left–right differences (left 20.3 mm; right 20.6 mm; P = 0.06). This study expands upon their conclusions by demonstrating not only the symmetric plafond radius of curvature in both tibial plafonds, but also the symmetry of the overall bony morphology. This enables us to state with greater confidence that contralateral templating is a suitable option for tibial plafond fractures.

Findings in these studies can be an asset to not only preoperative 3D virtual planning, but also to determine the quality of the postoperative reduction. In preoperative 3DVP, the unfractured 3D CT scan can be used as a template while reducing the fractured tibial plafond. To check the quality of the postoperative reduction, the CT scan of the unfractured contralateral tibial plafond and the postoperative CT-scan can be compared. In the future these findings might even be useful to shape 3D printed prosthetics for the tibial plafond in non-posttraumatic situations. Further research is necessary to prove this is a reliable comparison for pilon fractures.

### Limitations

Some limitations should be recognized. A first limitation of this study is based on the segmentation of the CT scans in low contrast areas. Within those regions, distinguishing bone and soft tissue is challenging, which may have introduced segmentation errors [38]. Low contrast areas were mostly located in the medial malleolus and the anterior tubercle of the tibial plafond, which may have contributed to the relatively large left–right differences in those regions (Fig. 3). To minimize the segmentation errors, all automatic segmentations were checked and corrected manually if needed.

Secondly, the accuracy of the left–right comparison was limited by the resolution of the CT images. The median left–right difference was less than the average voxel size of 0.791 × 0.791 × 0.8 mm. Hence, the observed differences may also be attributable to limitations in measurement accuracy. A smaller voxel size could increase the accuracy of the left–right comparison, but it would also increase the radiation dose [39]. However, for the purpose of 3DVP based on images with this resolution, we can conclude that the contralateral correspondence is sufficiently high, with deviations of less than a voxel.

Thirdly, this study solely focused on the comparison of the left and right tibial plafond. However, it is worth researching if this symmetry is affected by other parameters, such as a history of obesity or intensive weight bearing, which is not traceable for this study population.

## Conclusion

In conclusion, this study shows that the tibial plafond is highly symmetric with a median left–right difference of 0.57 mm. This indicates that left–right mirroring is a reliable method to optimize preoperative surgical reduction using 3DVP in pilon fractures.

## Data Availability

No datasets were generated or analysed during the current study.

## References

[1] Luo DT. Classifications in brief: Rüedi-Allgöwer classification of tibial plafond fractures. Clin Orthop Relat Res. 2017;475(7):1923–8. 28054323 10.1007/s11999-016-5219-z PMC5449320

[2] Mauffrey C, Vasario G, Battiston B, Lewis C, Beazley J, Seligson D. Tibial pilon fractures: a review of incidence, diagnosis, treatment, and complications. Acta Orthop Belg. 2011;77(4):432–40. 21954749

[3] Luo DT. Pilon fracture. In: StatPearls [Internet]. 2022. Available from: https://www.ncbi.nlm.nih.gov/books/NBK482176/.

[4] Zelle Boris BA. High-energy tibial pilon fractures: an instructional review. Int Orthop. 2019;43(8):1939–50. 31093715 10.1007/s00264-019-04344-8

[5] Cole PA. The pilon map: fracture lines and comminution zones in OTA/AO type 43C3 pilon fractures. J Orthop Trauma. 2013;27(7):152–6. 10.1097/BOT.0b013e318288a7e9 23360909

[6] Swords MP. High-energy Pilon fractures: role of external fixation in acute and definitive treatment. What are the indications and technique for primary ankle arthrodesis? Foot Ankle Clin. 2020;25(4):523–36. 33543715 10.1016/j.fcl.2020.08.005

[7] Bhattacharyya T. Complications associated with the posterolateral approach for pilon fractures. J Orthop Trauma. 2006;20(2):104–7. 16462562 10.1097/01.bot.0000201084.48037.5d

[8] Liu J. A systematic review of the role of surgical approaches on the outcomes of the tibia Pilon fracture. Foot Ankle Specialist. 2015;9(2):163–8. 26644032 10.1177/1938640015620637

[9] Hendrickx LAM. Incidence, predictors, and fracture mapping of (occult) posterior malleolar fractures associated with tibial shaft fractures. J Orthop Trauma. 2019;33(12):452. 10.1097/BOT.0000000000001605 31425412

[10] Williams TM. Factors affecting outcome in tibial plafond fractures. Clin Orthop Relat Res. 2004;423:93–8. 10.1097/01.blo.0000127922.90382.f4 15232432

[11] Stapleton JJ. Surgical treatment of tibial plafond fractures. Clin Podiatr Med Surg. 2014;31(4):547–64. 25281515 10.1016/j.cpm.2014.06.002

[12] Turow A. 3D mapping of scaphoid fractures and comminution. Skeletal Radiol. 2020;49(10):1633–47. 32417943 10.1007/s00256-020-03457-1

[13] Sandow M. The why, what, how and where of 3D imaging. J Hand Surg (Eur Vol). 2014;39(4):343–5. 24742741 10.1177/1753193414524137

[14] Yoshii Y. Computer-aided assessment of displacement and reduction of distal radius fractures. Diagnostics. 2021;11(4):719. 33919594 10.3390/diagnostics11040719 PMC8073711

[15] Hadad Matthew JM. Surgically relevant patterns in triplane fractures: a mapping study. J Bone Joint Surg Am. 2018;100(12):1039–46. 29916931 10.2106/JBJS.17.01279

[16] Prijs J. 3D virtual pre-operative planning may reduce the incidence of dorsal screw penetration in volar plating of intra-articular distal radius fractures. Eur J Trauma Emerg Surg. 2021;48(5):3911–21. 34623473 10.1007/s00068-021-01800-2 PMC9532324

[17] Merema BJ. The design, production and clinical application of 3D patient-specific implants with drilling guides for acetabular surgery. Injury. 2017;48(11):2540–7. 28899562 10.1016/j.injury.2017.08.059

[18] Leemhuis JF. Both-column acetabular fractures: does surgical approach vary based on using virtual 3D reconstructions? Diagnostics. 2023;13(9):1629. 37175020 10.3390/diagnostics13091629 PMC10178242

[19] Zhang H. Analysis for clinical effect of virtual windowing and poking reduction treatment for Schatzker III tibial plateau fracture based on 3D CT data. BioMed Res Int. 2015;2015:1. 10.1155/2015/231820 PMC4341848 25767804

[20] Moldovan F. Integration of Three-dimensional technologies in orthopedics: a tool for preoperative planning of tibial plateau fractures. Acta Inform Med. 2020;28(4):278–82. 33627930 10.5455/aim.2020.28.278-282 PMC7879455

[21] Chen S. Evaluation of the computer-assisted virtual surgical technology in preoperative planning for distal femoral fracture. Injury. 2020;51(2):443–51. 31771786 10.1016/j.injury.2019.10.085

[22] Mishra A. Virtual preoperative planning and 3D printing are valuable for the management of complex orthopaedic trauma. Chin J Traumatol. 2019;22(6):350–5. 31668700 10.1016/j.cjtee.2019.07.006 PMC6921216

[23] Maini L. Evaluation of accuracy of virtual surgical planning for patient-specific pre-contoured plate in acetabular fracture fixation. Arch Orthop Trauma Surg. 2018;138(4):495–504. 29368178 10.1007/s00402-018-2868-2

[24] Chen KK. Accuracy of virtual surgical planning in treatment of temporomandibular joint ankylosis using distraction osteogenesis: comparison of planned and actual results. J Oral Maxillofac Surg. 2018;76(11):1–2422. 30092217 10.1016/j.joms.2018.07.003

[25] Assink N. Does 3D-assisted surgery of tibial plateau fractures improve surgical and patient outcome? A systematic review of 1074 patients. Eur J Trauma Emerg Surg. 2021;48(3):1737–49. 34463771 10.1007/s00068-021-01773-2 PMC9192447

[26] Auerbach BM. Limb bone bilateral asymmetry: variability and commonality among modern humans. J Hum Evol. 2006;50(2):203–18. 16310833 10.1016/j.jhevol.2005.09.004

[27] Radzi S. Assessing the bilateral geometrical differences of the tibia–are they the same? Med Eng Phys. 2014;36(12):1618–25. 25271192 10.1016/j.medengphy.2014.09.007

[28] Tümer NN. Three-dimensional analysis of shape variations and symmetry of the fibula, tibia, calcaneus and talus. J Anat. 2019;234(1):132–44. 30393864 10.1111/joa.12900 PMC6284442

[29] Gabrielli AS. Bilateral symmetry, sex differences, and primary shape factors in ankle and hindfoot bone morphology. Foot Ankle Orthop. 2020;5(1):2473011420908796. 35097367 10.1177/2473011420908796 PMC8697112

[30] Melinska AU. Statistical, morphometric, anatomical shape model (Atlas) of calcaneus. PLoS One. 2015;10(8):e0134603. 10.1371/journal.pone.0134603 PMC4536012 26270812

[31] Li X. H-DenseUNet: hybrid densely connected UNet for liver and tumor segmentation from CT volumes. IEEE Trans Med Imag. 2018;37(12):2663–74. 10.1109/TMI.2018.2845918 29994201

[32] Myronenko A. Point set registration: coherent point drift. IEEE Trans Pattern Anal Mach Intell. 2010;32(12):2262–75. 20975122 10.1109/TPAMI.2010.46

[33] Chen H. A robust and semi-automatic quantitative measurement of patellofemoral instability based on four dimensional computed tomography. Med Eng Phys. 2020;78:29–38. 32115353 10.1016/j.medengphy.2020.01.012

[34] van der Gaast N. The symmetry of the left and right tibial plateau: a comparison of 200 tibial plateaus. Eur J Trauma Emerg Surg. 2022;49(1):69–74. 35829733 10.1007/s00068-022-02043-5 PMC9925587

[35] Letta C. Quantification of contralateral differences of the scaphoid: a comparison of bone geometry in three dimensions. Anat Res Int. 2014;2014:904275. 10.1155/2014/904275 PMC3942206 24715983

[36] Islam KK. Symmetry analysis of talus bone: a geometric morphometric approach. Bone Joint Res. 2014;3(5):139–45. 24802391 10.1302/2046-3758.35.2000264 PMC4037882

[37] Kellam PJ. Symmetry and reliability of the anterior distal tibial angle and plafond radius of curvature. Injury. 2020;51(10):2309–15. 32660695 10.1016/j.injury.2020.07.023

[38] Fu YY. Automatic and hierarchical segmentation of the human skeleton in CT images. Phys Med Biol. 2017;62(7):2812–33. 28195561 10.1088/1361-6560/aa6055

[39] Dach E. Impact of voxel size and scan time on the accuracy of three-dimensional radiological imaging data from cone-beam computed tomography. J Cranio-Maxillofac Surg. 2018;46(12):2190–6. 10.1016/j.jcms.2018.09.002 30318325

